# Perceptions of Stalking: Examining Perceivers’ Country of Origin, Perpetrator-Target Prior Relationship, and the Mediating Effect of Victim Responsibility

**DOI:** 10.1177/08862605211042601

**Published:** 2021-09-07

**Authors:** Kai Li Chung, Lorraine Sheridan

**Affiliations:** 1 University of Reading Malaysia, Nusajaya, Johor, Malaysia; 2 Curtin University, Bentley, WA, Australia

**Keywords:** stalking, harassment, domestic violence, perceptions of violence, legal intervention, cultural contexts

## Abstract

Research in stalking perceptions has shown certain relational biases, in which people tend to view ex-partner stalkers to be less dangerous than stranger or acquaintance stalkers. These findings are in direct contrast to those of real-life cases whereby ex-partner stalkers pose a greater threat. In addition, although stalking is recognized as a global social problem, most studies have been based on samples drawn from Western, educated, industrialized, rich, and democratic countries. The current study examined whether the prior relationship between the stalking perpetrator and target influences people’s perceptions of stalking and whether cross-national differences exist between participants based in Malaysia (where there is currently no law that criminalizes stalking) and England (where stalking has been outlawed since 1997). In a 3 × 2 between-subjects design, 294 Malaysian participants and 170 English participants were presented with a vignette describing a stalking scenario in which the perpetrator was depicted as a stranger, acquaintance, or ex-partner. Participants judged the extent to which the perpetrator’s behavior constitutes stalking; necessitates police intervention; would cause the victim alarm or personal distress; would cause the victim to fear the use of violence; and can be attributed to encouragement on the part of the victim. Results showed that typical relational biases existed in both samples, but Malaysian participants were less likely than their English counterparts to label any harassing scenario as serious. Perceptions of victim responsibility were found to mediate the effect of prior relationship and nationality on participants’ perceptions. The findings point to the urgency of better cross-cultural understanding of harassment behavior as well as legislations against stalking.

## Introduction

### Stalking Victimization and Perpetration

Unlike most crimes, stalking is not a single act, but a series of behaviors carried out over a period of time (Spitzberg & Cupach, 2007). Behaviors that do not break the law and are seemingly harmless when performed separately (e.g., phone calls, gift-giving, or texting) can be regarded as threatening when they escalate in frequency, duration, and intensity (Sinclair & Frieze, 2000). As such, stalking is easy to commit, but difficult to define and prosecute, in part because people vary in their judgments of how acceptable various intrusive behaviors are (Sheridan et al., 2019). While there is no single legal definition of stalking, the term generally refers to *a pattern of unwanted and repeated attention, harassment, contact, or any other course of conduct that is intentionally directed at a specific person or group that would cause a reasonable person to feel fearful or threatened* (Spitzberg & Cupach, 2007).

Stalking is known to be a widespread phenomenon around the world. While it is challenging to obtain accurate data on the prevalence of stalking due to inconsistencies in definitions, estimates of lifetime prevalence are generally similar across Western countries, including the United Kingdom, United States, and Canada, ranging between 7% and 36% in females, and 2% and 29% in males (see review by Spitzberg & Cupach, 2014). The adverse physical, psychological, social, and financial impacts of stalking on victims and their close others cannot be underestimated (Morewitz, 2003). A number of studies have reported an elevated risk of negative mental health outcomes, such as depression and post-traumatic stress (Bailey & Morris, 2018). Earlier research has shown that stalking often precedes fatal or near fatal violence (McFarlane et al., 1999). There are also considerable economic consequences, which may be attributed to productivity loss, property damage, medical treatment, and legal services (Peterson et al., 2018).

It appears that there is a high rate of self-identified stalking victimization. Due to the widespread nature of stalking and its negative consequences, researchers have sought to identify factors that may predict perpetration of stalking and help explain the behavior (Ménard & Pincus, 2012). Psychopathology has been referred to as a driving cause of stalking perpetration in some cases, and other reported nonclinical risk factors include childhood trauma, attachment anxiety, and personality characteristics (Dye & Davis, 2003; Nijdam-Jones et al., 2018). Higher rates of substance use have also been associated with increases in violent behavior and recidivism among stalking offenders (Rosenfeld, 2004). Understanding the link between motivations underlying the offending behavior has clinical utility, but it also raises awareness of stalking among criminal justice officials, victim service professionals, and the general public.

### Perceptions of Stalking

One particular area that has received considerable research attention is perceptions of stalking. Much empirical work has sought to identify personal and situational factors that influence people’s perceptions of stalking; prior relationship between the perpetrator and the victim is one such factor that has repeatedly shown an influence (Scott et al., 2014; Scott & Sheridan, 2011). The methodology in perception research typically involves manipulating stalking vignettes (i.e., scenarios that portray a particular pattern of conduct that may or may not constitute stalking) to assess individuals’ judgments of case severity as well as pursuer and victim culpability (for a review see Scott, 2020). It has been demonstrated that the greater the degree of prior intimacy between the stalker and the victim, the less likely people are to view a harassing situation as being serious. For instance, Scott and Sheridan (2011) examined three relational subtypes of stalkers (stranger, acquaintance, and ex-partner) and found that United Kingdom university students were more likely to judge harassing behavior as constituting stalking and call for police intervention and/or criminal charges when the behavior was performed by a stranger as opposed to an ex-partner. Participants in this study also tended to believe that a target would experience more alarm or distress when the harasser and target were portrayed as strangers. However, such perceptions often do not reflect the reality that stalkers are more likely to be ex-partners than strangers or acquaintances, and that ex-partner stalkers are often more persistent and dangerous than stranger or acquaintance stalkers (McEwan et al., 2009; Spitzberg, 2002). In addition, in a study by Sheridan and Roberts (2011), it was found that an abusive prior relationship between the victim and the stalker predicted physical assault.

A body of research (Cass & Mallicoat, 2015; Cass & Rosay, 2012) has also demonstrated that the nature of the perpetrator-victim prior relationship influences people’s perceptions of the criminal justice process in stalking cases. In these studies, university students tended to believe that relationship status would impact victim reporting as well as authorities’ arrest and investigative decisions. The bias toward judging stalking situations as less serious and victims as more responsible in cases where perpetrators are ex-partners as opposed to strangers or acquaintances has been shown to exist even among police officers. For example, Sheridan et al. (2016a) found that police officers had a higher tendency to consider harassment behavior as stalking, requiring police involvement, and causing the victim alarm and fear of violence when the perpetrator was a stranger instead of an ex-partner. Notably, policing experience played a role; officers with direct experience with stalking-related investigations (Weller et al., 2013) and specialist officers who had prior training in interpersonal violence cases (Scott et al., 2013) were generally less likely than nonexperienced and nonspecialist officers to blame the victim for such situations. Such relational biases resonate with findings from the violent crime literature, particularly in relation to victim-blaming attributions. According to a review by van der Bruggen and Grubb (2014), earlier works have indicated that stranger rape victims are blamed more than acquaintance rape victims, but in more recent studies it appears that victims are apportioned greater blame in date or acquaintance rape cases than in stranger rape cases. The inconsistent findings are likely due to the use of different manipulations in the vignette methodology. The impact of marital rape on victims, on the other hand, is consistently minimized in the literature. It can generally be concluded that the better the perpetrator and victim know each other, the higher the likelihood that blame will be assigned to victims of violence.

Misperceptions the public hold about stalking behavior, if left unaddressed, may lead to a lack of demand for policy and social change. The fact that the common misperception that ex-partner stalkers present a lesser threat to their victims’ personal safety than acquaintance and stranger stalkers is apparent among police officers is problematic, as this may impact their decision-making about the seriousness of stalking cases. It is thus critical to identify contexts that contribute to common misperceptions so that such misperceptions can be challenged through appropriate awareness and training programs.

### Stalking Across Cultures

While stalking is recognized as a global issue, the majority of prior research has been based on samples drawn from Western, educated, industrialized, rich, and democratic (WEIRD) countries (Henrich et al., 2010), where stalking is typically considered a criminal offence. Studies conducted within the Asia region remains limited, except in Japan (Chapman & Spitzberg, 2003), Singapore (Sheridan et al., 2019), Hong Kong, and China (Chan & Sheridan, 2020).

The very few studies conducted in Asia have proposed that cultural values and practices may have an influence on people’s perceptions of stalking. For example, in the study by Chapman and Spitzberg (2003), more American than Japanese students who had been “persistently pursued” perceived themselves as being subjected to stalking, but more Japanese than Americans considered the intrusive behaviors as threatening. It was put forward that the difference between the collectivism of Japanese society and the individualism of American society may play a role; being group-centered, the Japanese may have a preference to avoid conflict and hence be more hesitant to report such intrusive behaviors.

According to a study conducted among young women across 12 countries (Armenia, Australia, England, Egypt, Finland, India, Indonesia, Italy, Japan, Portugal, Scotland, and Trinidad), people’s reported experience of intrusive behavior varied depending on national levels of gender empowerment—a measure of women’s societal power (Sheridan et al., 2016b). It was found that women from countries with lower gender empowerment scores (e.g., Egypt, Indonesia) reported having experienced arguably more sinister intrusions (e.g., forced sexual contact, death threats, being spied upon), whereas women from countries with higher gender empowerment and individualism scores reported having experienced activities that are typically seen as relatively innocuous (e.g., being offered a drink by a stranger, being asked for casual sex at a social event). Such findings were in line with literature (Archer, 2006) that suggests that women from collectivists societies tend to have lower societal power, making them more vulnerable to male-perpetrated violence.

It seems evident that cross-cultural and/or cross-national variations in people’s perceptions and experiences of stalking will exist, and as such, more cross-cultural data should be collected to gain more nuanced insights. The present study examined perceptions of stalking among student and community samples in Malaysia (where there is currently no law that criminalizes stalking) and England (where stalking has been outlawed since 1997).

### Stalking in Malaysia and England

Current data on the prevalence and incidence of stalking in Malaysia are compiled largely by nongovernmental charitable organizations that work with abuse victims. According to estimates by the Women’s Aid Organisation in Malaysia, approximately 26% of 900,000 domestic violence survivors in Malaysia have been stalked by their abusers (Women’s Aid Organisation, 2013). This reported figure, while anecdotal, appears to be consistent with statistics in other countries (Spitzberg & Cupach, 2014).

While many countries have either specific anti-stalking laws (e.g., Canada, Japan) or incorporated stalking into their respective criminal codes (e.g., Germany, India), stalking has yet to be made a crime in Malaysia. For this reason, there is little that the authorities can do when a victim of stalking makes a police report, even if an investigation takes place. The 2017 amendments to the Domestic Violence Act 1994, which have included improved protection orders for victims and a widened definition of domestic violence, do offer some form of protection to stalking victims. Under this act, committing any form of violence against a spouse, a former spouse, or a family member counts as domestic violence, which is an offence. However, this act does not apply if the perpetrator is not related or married to the victim, or if the victim is unable to prove obvious injuries.

In England and Wales, the Protection from Harassment Act was introduced in 1997 to recognize stalking as a crime, but “stalking” was not specifically named in said legislation. The Protection of Freedoms Act (2012) extended this earlier legislation to include two new offences, namely “stalking” and “stalking involving fear of violence,” purportedly to distinguish between a behavior that constitutes a low-level harassment offence and a higher-level offence that causes fear of violence or serious distress to the victim.

A recent report by the Crown Prosecution Service (2020) in England and Wales revealed that the number of recorded charges in the last two years more than doubled the number five years previously, with most cases committed by ex-partners. It was postulated that the rise in number of charges is partly because police and prosecutors are better able to recognize stalking as a part of a wider pattern of domestic abuse. This seems to suggest that better awareness of stalking offences directly impacts prevention and intervention efforts.

## The Present Study

This study examined whether the prior relationship between the stalking perpetrator and victim (stranger, acquaintance, or ex-partner) influences people’s perception of whether the perpetrator’s behavior constitutes stalking; necessitates police intervention; causes the victim alarm or personal distress; causes the victim to fear the use of violence; and can be attributed to encouragement on the part of the victim. This study was particularly focused on whether cross-national differences in perceptions exist between participants based in Malaysia and England, and, if they do, potential explanations for this.

Additionally, this study explored the extent to which attribution of victim responsibility mediates the effect of prior relationship and nationality on perceptions of whether the perpetrator’s behavior constitutes stalking; necessitates police intervention; causes the victim alarm or personal distress; and causes the victim to fear the use of violence. Only one earlier work (Sheridan et al., 2016a) examined whether victim responsibility was a significant mediator of perceptions of stalking. This study was conducted using a police sample and found that target responsibility partly mediated officer perceptions. The present study explored this mediation role within a general population sample in order to examine whether this often-inferred relationship can be observed at a statistically significant level.

The present study fills a gap by including empirical data from an Asian country, contributing to the existing literature in a significant way. It was conducted in Malaysia, a Southeast Asian country with a complex multiracial Asian population. This study provides insights into how acceptable stalking is considered in different regions, particularly given that Malaysia inherited its common law from the United Kingdom.

## Methods

### Participants

A total of 574 participants submitted their responses, but only responses with a completion rate of at least 80% were included in the analyses (464, or 80.84%). Malaysian participants were recruited from student and community samples using opportunity sampling. The online study was advertised to students on the University of Reading Malaysia campus using the University’s research participation pool and on the social media networks of the researchers. The data included 294 Malaysians (104 males, 189 females, and one preferred not to say) aged 18 to 71 (*M* = 28.73, *SD* = 11.71). Most were ethnic Chinese (48.0%), followed by ethnic Malay (38.8%), ethnic Indian (5.8%), and other ethnicities (6.1%), while 1.4% preferred not to say. More than half (57.5%) of the sample were single at the time of the study, 12.2% were in a relationship, 28.6% were married, 0.3% were divorced, 1.0% were widowed, and 0.3% classified their marital status as others. Most participants (75.5%) had no children. About half of the Malaysian participants (53.4%) had attained at least a bachelor’s degree.

English participants consisted of primarily undergraduate psychology students on the United Kingdom campus, which is situated in Southern England. Students were recruited using the University of Reading Malaysia research participation pool. There were 170 English participants (16 males and 154 females) aged 18 to 66 (M = 21.58, SD = 7.85). The majority of the sample were White (71.2%), 14.7% were Asian British, 2.9% were Black British, 8.8% were of other ethnic backgrounds, while 2.4% preferred not to say. About half (51.8%) were single at the time of the study, 41.2% were in a relationship, 6.5% were married, and 0.6% were divorced. Almost all participants (94.7%) did not have children. Less than one third (28.3%) of the English participants had completed at least a bachelor’s degree, but the majority (70.6%) had further education (e.g., A-levels) or diplomas as their highest qualification.

### Measures and Procedure

Data was collected as part of a larger project examining dispositional and contextual factors that may contribute to perceptions of stalking. This was an anonymous study administered via an online platform, Qualtrics. The study was advertised as a “Perceptions of Interpersonal Behavior” study. Informed consent was obtained. Following the research paradigm by Scott et al. (2013), participants’ perceptions of stalking were then examined using a written one-paragraph vignette. There were three versions of the vignette, representing the different degree of prior intimacy between the perpetrator and the victim: stranger, acquaintance, and ex-partner. All three versions described the same stalking scenario; the stranger vignette is presented as follows:

Liza first met Adam when she visited the estate agents where he works to renew the lease on her apartment. As Liza was leaving the office Adam asked if she would like to join him for lunch. Liza thanked him for the offer, but declined. During the 3 months that followed, Adam sent Liza between 5 and 10 text messages a day, many of these messages asking why she was not interested in him. Adam also approached Liza on her way to work and telephoned her at home. Liza asked Adam to stop calling her, but he continued to call her regularly. In the end Liza disconnected the phone and Adam left several messages blaming her for what was happening. Most recently, Adam arrived at Liza’s home soon after she returned from work. Liza pretended that she was out.

In the acquaintance condition Liza and Adam had worked together for three months when he invited her for lunch. In the ex-partner condition, Liza and Adam had been in a romantic relationship, but she ended it when she realized they wanted different things from the relationship. All participants were randomly assigned into one of the three conditions.

Each vignette was followed by five 11-point Likert-type statements to measure participants’ perceptions of stalking. The statements are as follows:

1. To what extent does Adam’s behavior constitute stalking? (“Definitely not stalking” to “Definitely stalking”)
2. To what extent does Adam’s behavior necessitate police intervention? (“Not at all necessary” to “Extremely necessary”)
3. Do you think Adam’s behavior will cause Liza alarm or personal distress? (“Definitely not” to “Definitely”)
4. Do you think Adam’s behavior will cause Liza to fear that he will use violence against her? (“Definitely not” to “Definitely”)
5. To what extent is Liza responsible for encouraging Adam’s behavior? (“Not at all responsible” to “Totally responsible”)

Participants then completed a demographic information questionnaire that comprised questions about nationality, age, gender, ethnic background, marital status, number of children, and level of education. Participants were provided with a debrief sheet upon completion. This study received ethical approval from the University of Reading Malaysia Research Ethics Committee.

## Results

A 3(prior relationship: stranger, acquaintance, ex-partner) × 2(nationality: Malaysian, English) MANOVA showed significant main effects of prior relationship, *F*(5, 458) = 4.35, *p* < .001, Wilks’ Λ = .91, partial η^2^ = .05 and nationality *F*(5, 458) = 30.26, *p* < .001, Wilks’ Λ = .75, partial η^2^ = .25. Table 1 shows descriptive statistics for all five perception scale items, whereas Table 2 shows *F* ratios for the perception items by prior relationship conditions and nationality. There was a significant main effect of prior relationship on all individual perception scale items. A Tukey post hoc test showed that overall participants were more likely to believe that the harassing behavior constituted stalking, that police intervention was necessary, that the behavior would cause the victim alarm or distress and fear of violence, and that the victim was less responsible for the behavior when the perpetrator was portrayed as a stranger or acquaintance rather than an ex-partner. There was also a significant main effect of nationality on all individual perception scale items except for fear of violence, *p* = .68. Compared to Malaysians, English participants were more likely to consider the perpetrator’s behavior to constitute stalking, necessitate police intervention, cause the victim alarm or personal distress, but less likely to think the victim was responsible for encouraging the perpetrator’s behavior. There was no significant interaction effect between prior relationship and nationality on the five perception scale items, *F*(5, 458) = 1.58, *p* = .11, Wilks’ Λ = .97, partial η^2^ = .02.

**Table 1.** Means and Standard Deviations for the Five Perception Scale Items by Prior Relationship Conditions and Nationality.

Condition	*M* (*SD*)				
	Stalking	Intervention	Alarm	Violence	Responsibility
Overall					
Stranger	8.43 (2.20)	7.41 (2.44)	8.96 (1.72)	7.86 (2.12)	1.96 (2.65)
Acquaintance	8.22 (2.12)	7.28 (2.39)	8.99 (1.57)	7.99 (2.10)	2.01 (2.70)
Ex-partner	7.23 (2.78)	5.97 (3.17)	8.42 (2.12)	7.25 (2.46)	3.47 (3.11)
Malaysian					
Stranger	8.15 (2.41)	7.05 (2.74)	8.75 (1.94)	7.92 (2.27)	2.58 (2.79)
Acquaintance	7.71 (2.44)	6.98 (2.66)	8.80 (1.70)	8.09 (2.34)	2.86 (2.92)
Ex-partner	6.57 (3.10)	5.24 (3.40)	8.03 (2.43)	7.20 (2.73)	4.74 (3.07)
Total	7.47 (2.75)	6.41 (3.07)	8.52 (2.07)	7.73 (2.48)	3.40 (3.07)
English					
Stranger	8.93 (1.67)	8.05 (1.57)	9.35 (1.17)	7.76 (1.83)	0.84 (1.94)
Acquaintance	9.05 (1.03)	7.78 (1.79)	9.29 (1.27)	7.84 (1.63)	0.64 (1.52)
Ex-partner	8.39 (1.56)	7.26 (2.22)	9.11 (1.16)	7.33 (1.89)	1.25 (1.52)
Total	8.79 (1.46)	7.69 (2.22)	9.25 (1.20)	7.65 (1.79)	0.91 (1.68)

**Table 2.** Multivariate and Univariate Analyses of Variance *F* Ratios for the Five Perception Scale Items by Prior Relationship Conditions and Nationality.

	MANOVA	ANOVA				
	*F*	*F*				
Condition		Stalking	Intervention	Alarm	Violence	Responsibility
Relationship	4.35**	8.93**	10.50**	3.45*	4.01*	11.92**
Nationality	30.26**	34.94**	25.56**	17.55**	0.17	103.17**
Relationship × Nationality	1.58	1.83	2.27	1.09	0.28	4.57*

*Note.* *p < .05; **p < .01.

**Figure 1.**
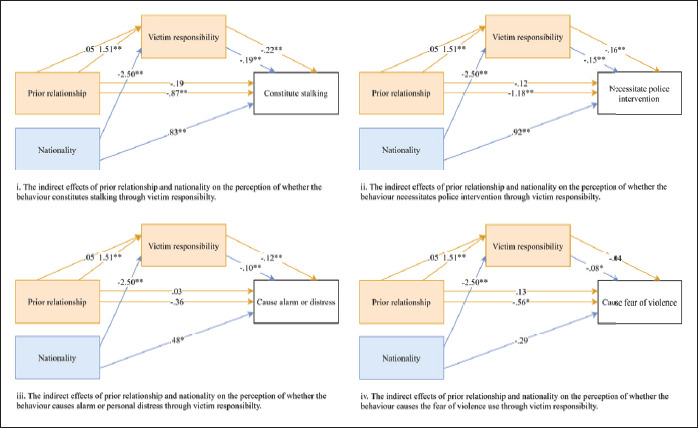
Models representing the mediated effect of prior relationship and nationality on the five perception scale items through victim responsibility.

*Note.* *p < .05; **p < .01.

Analyses using the PROCESS macro model 4 were conducted to determine whether attribution of victim responsibility mediated the effect of prior relationship and nationality on perceptions of stalking. Figure 1 shows the mediation model diagrams for each perception item. As predicted, there were significant indirect effects of victim responsibility between prior relationship conditions on whether the behavior was judged to: constitute stalking, *indirect =* –.33, *SE* = .10, 95% CI [–.55, –.16], necessitate police intervention, *indirect =* –.25, *SE* = .10, 95% CI [–.46, –.09], and cause the victim alarm or distress *indirect =* –.18, *SE* = .06, 95% CI [–.32, –.08]. The indirect effect of victim responsibility between prior relationship conditions on the extent to which the behavior was perceived to cause the victim to fear violence was nonsignificant, *indirect =* –.06, *SE* = .06, 95% CI [–.18, .05]. There were also significant indirect effects of victim responsibility on nationality concerning whether the behavior was believed to: constitute stalking, *indirect =* .49, *SE* = .13, 95% CI [.25, .74], necessitate police intervention, *indirect =* .37, *SE* = .14, 95% CI [.09, .66], and cause the victim alarm or distress *indirect =* .25, *SE* = .08, 95% CI [.09, .42]. The indirect effect of victim responsibility between nationality on the extent to which the behavior was perceived to cause the victim to fear violence was nonsignificant, *indirect =* –.21, *SE* = .10, 95% CI [.01, .42].

## Discussion

The present study sought to examine the role of prior relationship between stalking perpetrator and victim in perceptions of a stalking scenario, within Malaysian and English populations. First, findings indicate that prior relationship between the perpetrator and victim of stalking influenced how participants responded on all five perception scale items. Participants of both nationalities were more likely to think the behavior constituted stalking, warranted police intervention, would result in the victim feeling alarm or distress, and to consider that the victim would fear the use of violence when the perpetrator was portrayed as a stranger or acquaintance to the victim rather than an ex-partner. Participants were also more likely to believe that the victim was responsible for the harassing behavior when the perpetrator and the victim were depicted as prior ex-intimates. The data are in line with existing findings that have demonstrated a robust relational bias (Scott et al., 2014; Scott & Sheridan, 2011).

Second, there were significant differences in perceptions between participants of both nationalities across conditions. Specifically, Malaysians were less likely than English participants to perceive the perpetrator’s behavior as stalking, requiring police intervention, and causing the victim alarm or personal distress. Malaysians were also more likely than English participants to judge the victim as responsible for encouraging the perpetrator’s behavior.

From these findings, it can be concluded that although the typical relational biases still existed in both samples, Malaysian participants were less likely than their English counterparts to label the harassing scenario as stalking. This indicates that Malaysians may underestimate the severity of stalking cases more so than English populations. Moreover, perceptions of victim responsibility mediated the effect of prior relationship and nationality on people’s perceptions of whether a harassing behavior by a perpetrator is considered stalking, requires police intervention, or causes distress in the victim. This suggests that perpetrator-victim relationship status and perceiver nationality cannot fully explain how people perceive harassment situations, and that a variety of other factors—some remaining unexplored—have an impact on blame attribution, which in turn predicts stalking perceptions. As outlined previously, this attribution of blame toward victims or victim-blaming phenomenon, whereby victims rather than the perpetrator are made to feel responsible when an assault occurs, is a key theme within the rape and domestic violence literature. It has previously been postulated as a factor that influences the perceived seriousness of stalking incidents (Boehnlein et al., 2020; Korkodeilou, 2014; Sheridan et al., 2016a).

According to Grubb and Turner (2012), victim-blaming tends to be perpetuated by a variety of cognitive and motivational biases, which could be a result of one’s personality disposition and social prescriptions. One theoretical explanation of this counterintuitive response to crime victims is the just world theory (Lerner, 1980), which refers to the tendency to believe that the world is a fair place and that “people get what they deserve and deserve what they get.” This perspective posits that negative victim perception occurs due to an overcompensation when judging a seemingly undeserving act; holding victims responsible for their misfortune helps observers regain their sense of control and restore congruence with the view that the world is just and orderly. In the case of stalking victimization, just world beliefs offer a justification as to why victims are harassed (i.e., they did not do enough to protect themselves, they precipitated or provoked their own victimization through their character or behavior, wittingly or not). This perspective cannot be as easily adopted when no previous history exists between perpetrator and target.

Hofstede’s (1980) cultural dimensions could be used to explain the differences observed in attitudes and perceptions held toward victims of stalking across the different countries. Malaysia is one of the Southeast Asian countries that has a complex multiracial Asian population, consisting of three main ethnic groups, namely Malay, Chinese, and Indian, as well as other indigenous groups. Cognitive schemas may very well be influenced by such factors. As per Hofstede’s theory (Hofstede, 2011), Malaysia is considered a collectivistic society with high power distance. People in such societies are more likely to accept and operate under a hierarchical structure, but they also tend to avoid situations that may endanger social harmony, through suppression of their own thoughts and feelings. Challenging governing authorities also tends not to be well-received in high power distance societies; this has profound consequences as the public will be less likely to lodge complaints against police inaction or misconduct. Experimental and survey studies have indicated that individuals with high power distance orientation, particularly those of Asian descent, tended to report lower rates of sexual harassment than people in low power distance cultures, suggesting that those who are likely to accept power differentials may be more tolerant of behaviors that count as harassment and perceive such behaviors as less severe (Kennedy & Gorzalka, 2002; Mishra & Stair, 2019).

Furthermore, akin to most violent crimes, stalking tends to be framed as a gendered crime. One point that is relevant in the context of Malaysia where violence against women is arguably prevalent in parts of the country is the traditional attitudes toward gender norms (Alam & Ilias, 2014; Endut et al., 2020). A strong adherence to traditional masculine norms and belief in gendered power dynamics are associated with higher likelihood of perpetration of violence against women (Willie et al., 2018). Further, it has been argued that individuals living in societies where men hold authority over women tend to adhere to ‘rape myths’ that encompass problematic stereotypical assumptions about the likely behavior of perpetrators and victims (Ward, 1995). However, as cultural dimensions were not actually measured in the current study, it is premature to assume that all individuals within Malaysia share the same values. Taken together, it is evident that individual attitudes toward harassment behaviors differ based on the perceiver’s country of origin, but it is arguable that the oversimplistic individualist-collectivist dichotomy that is often put forward as a cultural explanation of cross-national differences is inadequate.

Another theory that is central to the literature on victim-blaming is the attribution theory (Heider, 1958), which relates to the way in which people use available information to arrive at causal explanations for events that have occurred. Individual differences in attributional style can influence how people respond to harassment behaviors. Observers of stalking cases with the propensity to utilize an internal attribution are more likely to infer that the harassment incidents are due to personal factors such as traits, abilities, or physical characteristics of the victims. This requires corroboration; future work should expand the literature by examining the role of individual differences variables such as personality factors in predicting attitudes toward stalking victimization.

## Limitations

As mentioned, stalking is a crime that shows a gendered victimization pattern and as such, the vignettes included in this study involved a male perpetrator and a female target. Existing findings have found that perceptions of seriousness are greater when the behavior is perpetrated by a man rather than by a woman (Scott et al., 2019), although there is work that suggests that the actual harm of stalking on the victim is equally severe and therefore should be taken just as seriously (Strand & McEwan, 2012). The generalizability of this study’s findings is therefore limited. Given that sociocultural context influences gender role expectations, and that such expectations may result in differential treatment by the criminal justice system, future research in the Southeast Asia region should consider the role of both perpetrator and victim gender in stalking perception research.

There was also a significant difference in sample characteristics. For one, the English participants were significantly younger in age compared to the Malaysians and comprised mainly university students, whereas the Malaysian sample reflected a more diverse

demographic. It is, however, possible that the young age of English participants meant they have grown up in a cultural context that is more attentive to issues of gender equality and violence against women in general, hence the stronger inclination to label harassment behaviors as stalking incidents causing distress and needing intervention from law enforcement agencies. Further, in both samples there was a large gender imbalance, with significantly more females compared to males, which would presumably have implications for the interpretation of findings.

Arguably, the five perceptual items in the current study do not adequately capture the complexity of how people perceive interpersonal violence. More in-depth analyses of the perception constructs via qualitative designs would have been able to address this limitation. More recently, perception researchers have also proposed using more sophisticated designs such as videotaped vignettes to provide perceivers with more realistic and contextual information (van der Bruggen & Grubb, 2014). Adoption of these recommendations will allow for better construct and ecological validity.

## Conclusions and Future Directions

The current study offers an insight into perceptions of stalking among people from different legislations and cultural backgrounds. This is one of the few stalking perception studies that was not wholly based on samples drawn from WEIRD societies that have enacted anti-stalking laws.

The present findings highlight the need to improve criminal justice responses to victims of harassment and stalking. A recent review of the criminal justice system in the United States by Backes et al. (2020) has shown that police are generally unfamiliar with what behaviors constitute stalking and typically regarded intimate partner stalking as situations where victims could potentially “work it out” with the stalker. This is likely to be the case in Malaysia since there is currently no clear legislative framework to criminalize stalking. Charity organizations in Malaysia are lobbying for the introduction of anti-stalking laws. Given the significant negative effects in the lives of victims, it is hoped that stalking will soon be recognized as a crime, after which future work should focus on using a longitudinal study design to examine how far people’s stalking perceptions are shaped by policy and awareness. Perceptions of police officers would be particularly important, as they are the frontline workers who make arrest decisions pertaining to such incidents. To believe that ex-partner stalkers present a lower risk of violence and therefore require less police intervention is a misperception; a dismissive response or a disinclination to acknowledge the severity of stalking behavior may result in improper treatment of victims (van der Aa & Groenen, 2011). It should, however, be noted that the enactment of legislation does not equate to better police practice or knowledge of the issue of stalking (Taylor-Dunn et al., 2018). Clear policies and adequate training led by specialists in the field would ensure that the social problem of stalking victimization is taken seriously.

## Author Biographies

**Kai Li Chung**, PhD, is a lecturer in the School of Psychology and Clinical Language Sciences at University of Reading Malaysia. Her research interests fall under personality and forensic psychology. Her current work focuses on the role of aversive personality traits in interpersonal violence.

**Lorraine Sheridan**, PhD, is an associate professor in the School of Population Health at Curtin University and a forensic psychologist. She is recognized as a global expert on stalking. Her research interests are in cross-cultural investigations, the association between stalking and sex offending, young stalkers, and the effects of psychological versus physical damage on stalking victims.
